# An H2A histone isotype regulates estrogen receptor target genes by mediating enhancer-promoter-3′-UTR interactions in breast cancer cells

**DOI:** 10.1093/nar/gkt1341

**Published:** 2013-12-25

**Authors:** Chia-Hsin Su, Tsai-Yu Tzeng, Ching Cheng, Ming-Ta Hsu

**Affiliations:** ^1^Institute of Biochemistry and Molecular Biology, School of Life Science, National Yang-Ming University, Taipei 11221, Taiwan, Republic of China, ^2^VYM Genome Research Center, National Yang-Ming University, University System of Taiwan, Taipei 11221, Taiwan, Republic of China and ^3^Chien-Tien Hsu Cancer Research Foundation, Taipei 11221, Taiwan, Republic of China

## Abstract

A replication-dependent histone H2A isotype, H2ac, is upregulated in MCF-7 cells and in estrogen receptor-positive clinical breast cancer tissues. Cellular depletion of this H2A isotype leads to defective estrogen signaling, loss of cell proliferation and cell cycle arrest at G0/G1 phase. H2ac mediates regulation of estrogen receptor target genes, particularly *BCL2* and *c-MYC*, by recruiting estrogen receptor alpha through its HAR domain and facilitating the formation of a chromatin loop between the promoter, enhancer and 3′-untranslated region of the respective genes. These findings reveal a new role for histone isotypes in the regulation of gene expression in cancer cells, and suggest that these molecules may be targeted for anti-cancer drug discovery.

## INTRODUCTION

Eukaryotic DNA is packaged into chromatin through association with histones (1). The basic chromatin unit, the nucleosome core particle, consists of 146-base pair units wrapped around a histone octamer composed of two molecules of each of the core histone proteins, H2A, H2B, H3 and H4 (2). In addition, the linker histone, H1, associates with linker DNA that is present between the nucleosomes, and produces a higher-order structure that stabilizes the 30-nm chromatin fiber (3).

The variants and modifications of the histones give rise to a complex and diverse chromatin structure that is necessary for proper nuclear function and epigenetic inheritance. Accordingly, in the recent years, N-terminal tail-modifications of histones, including methylation, acetylation and phosphorylation, have been extensively studied. These modifications regulate cell proliferation, growth and differentiation by altering chromatin structure and gene activity (4–6). Histones are a family of sequence variants encoded by distinct genes. Histone variants can be divided in two groups based on their characteristic modes of expression. The largest group, replication-dependent histones, is cell cycle regulated, and their expression is replication-dependent. The second group, replacement variants, exhibits replication-independent expression throughout the cell cycle. These different histone variants contribute to the creation of distinct or unique nucleosomal architectures. Through the resulting heterogeneity in nucleosomal architecture, a wide range of nuclear functions are regulated. Special replication-independent histones, such as H1.2, regulate global nucleosome spacing and control the expression of cell cycle genes, whereas macroH2A, which is associated with inactive X chromosome, is involved in the regulation of gene expression programs during cellular differentiation and embryonic development. H2A.Z has been implicated in the transcriptional regulation and gene silencing that involves modulation of the nucleosome array (7–11).

Sixteen replication-dependent histone H2A genes have been identified in the human genome (12). These genes are distributed across at least three clusters, with 12 located in the *Hist1* cluster on chromosome 6, 3 in the *Hist2* cluster and 1 in *Hist3* cluster on chromosome 1 (12). These replication-dependent histone H2A isotypes differ in their coding sequences, but are similar in their amino acid sequences. Interestingly, HIST1H2AC and HIST1H2AA differ from other canonical H2A histones in the *Hist1* cluster by an amino acid substitution at position 16—a change from threonine to serine. Previously, we noticed overexpression of *Hist1H2AC* and absence of *Hist1H2AA* in MCF-7 cells in comparison with non-tumorigenic MCF-10F cells (13). Here, we show that the H2A subtype, HIST1H2AC (abbreviated as H2ac from this point onward), which contains an HAR domain, is specifically expressed in estrogen receptor-positive (ER+) breast cancer tissues, but not in estrogen receptor-negative (ER−) and normal tissues. To examine the role of this H2ac in breast tumorigenesis, we analyzed the regulation of estrogen receptor (ER) target genes following knockdown of this gene and overexpression of its HAR domain mutants in MCF-7 cells. Our results showed that H2ac acts as a master regulator of estrogen receptor alpha (ERα)-dependent gene expression. This process occurred by recruiting activator ERα and mediating an interaction between the promoter, enhancer and 3′-untranslated region (3′-UTR) of the respective genes. The upregulation of oncogenes by H2ac through the recruitment of an activator is a new mechanism of tumorigenesis, and may be targeted for disease intervention.

## MATERIALS AND METHODS

### Cell culture and transfection

MCF-7 cells were grown in RPMI 1640 medium supplemented with 10% fetal bovine serum (FBS). MCF-10F and MCF-10A cells were cultured in a 1:1 mixture of Dulbecco's modified Eagle's medium and F12 media containing 20 ng/ml epidermal growth factor, 100 ng/ml cholera toxin, 0.01 mg/ml insulin, 500 ng/ml hydrocortisone and 5% horse serum (Sigma). For experiments involving E2 treatment, MCF-7 cells were grown in RPMI 1640 medium without phenol red (Gibco), supplemented with 5% charcoal-dextran–treated FBS for at least 3 days. 17β-Estradiol, tamoxifen (TAM) and ICI 182780 (Sigma) were used at concentrations of 10, 1 and 100 nM, respectively, unless otherwise stated. Cells were transfected using LipofectAMINETM RNAiMAX (for siRNA) or Lipofectamine® LTX with Plus™ Reagent (Invitrogen) according to manufacturer’s instructions.

### Flow cytometry

For flow cytometry, cells were harvested by trypsinization, centrifuged and resuspended in phosphate buffered saline (PBS). This was followed by fixation by adding 90% methanol maintained at −20°C. The fixed cells were washed with PBS, resuspended in 4 mM sodium citrate containing 30 U/ml RNAase A, 0.1% Triton X-100 and 50 μg/ml propidium iodide, and incubated for 10 min at 37°C. Cells were analyzed using a FACScan flow cytometry system.

### siRNA knockdown and real-time quantitative polymerase chain reaction analysis

For *H2ac*, and *LSD1* gene knockdown, MCF-7 cells were transfected with *H2ac* siRNA duplex (a mixture of equimolar concentrations of 5′-CUGCUAGGCCGGGUGACCA-3′ and 5′-UGGUCACCCGGCCUAGCAG-3′), *or LSD1* siRNA duplex (a mixture of equal molar concentrations of 5′-CAAUUAGAAGCACCUUAUA-3′ and 5′- UAUAAGGUGCUUCUAAUUG-3′) using LipofectAMINETM RNAiMAX (Invitrogen). The siRNAs were designed by Sigma-Aldrich. Quantitative polymerase chain reaction (qPCR) was performed using SYBR Green dye as a probe on a Roche Applied Science LightCycler® 2.0 Real-Time PCR System. All reactions were performed in triplicate, using SYBR Green Master Mix (Sigma) and 20 M each of forward and reverse primers according to the manufacturer’s recommended thermocycling conditions. The calculated quantity of the target gene was divided by the average sample quantity of the appropriate housekeeping genes, either RPS13 or 18s rRNA, to obtain the relative levels of gene expression. Primer sequences are described in Supplementary Table S1, presented as part of the Supplemental Material.

### Oncomine analyses

We obtained H2 expression data for clinical breast cancer samples from the TCGA Web site (http://tcga-data.nci.nih.gov/). These expression data were gathered on two individual microarray platforms, including TCGA Breast and Perou Breast. Statistical analysis of the differences in H2ac expression between these tissues was performed using Oncomine algorithms, which allowed multiple comparisons between various studies (14,15).

### Immunohistochemistry

Immunohistochemical staining of H2ac protein was performed using breast tissue array, BR1503b (US Biomax). Tissue sections were deparaffinized, rehydrated, soaked in antigen retrieval buffer (pH 9.0, Dako) and heated in a microwave oven for 10 min twice under defrosting conditions. The sections were then washed with PBS, and the endogenous peroxidase activity was quenched by applying 3% hydrogen peroxide for 5 min. This was followed by incubating with magic blocking reagent [1% Cold Water Fish Skin Gelatin (EMS) and 5% bovine serum albumin in PBS] for 1 h at room temperature. Subsequently, the sections were washed once with PBS for 10 min and incubated with H2AC antibody (SAB1303096, Sigma) at 4°C for 16–18 h. Following three washes with PBS for 10 min each, tissue sections were incubated with biotinylated secondary antibody for 1 h at room temperature. VECTASTAIN R Elite ABC kit (vector laboratory) and NovaRed TM HRP substrate kit (Vector Laboratory) were used for detection. The sections were counterstained with hematoxylin for 30 s, dehydrated, cleared in xylene and mounted.

### RNA extraction and Affymetrix microarray analysis

Total RNA was extracted using an RNeasy® Mini Kit (QIAGEN) following manufacturer’s protocols. Affymetrix microarray analysis was performed using a Human U133 plus 2.0 (Affymetrix) chip. The detailed methods used for RNA quality assessment, sample labeling, hybridization and expression analysis are described in the manual that accompanies the Affymetrix Microarray Kit.

### Microarray data

All Affymetrix data were MIAME compliant, and the raw data have been deposited in a MIAME compliant database, GEO. The microarray data set has been deposited at the NCBI GEO Web site (GEO accession number GSE39786).

### Immunoprecipitation

HEK-293T cells were transiently transfected by the liposome method, and whole cell extracts were prepared 36–48 h after transfection. The extracts were incubated with anti-HA agarose (Sigma) at 4°C for 2–4 h. Immunoprecipitated proteins were resolved by 12% sodium dodecyl sulphate-polyacrylamide gel electrophoresis (SDS-PAGE), transferred onto PVDF membranes and probed using various antibodies.

### *In vitro* pull-down assay

Flag- and HA-tagged proteins were created using an EasyXpress Protein Synthesis kit (QIAGEN) according to the manufacturer’s protocol. Briefly, *in vitro*-translated proteins were incubated with 10 ml of EZviewTM anti-HA affinity gel (Sigma, St. Louis, MO, USA) overnight at 4°C. The beads were retrieved and washed five times in 500 µl of PBS containing 0.5% NP-40 and boiled in 20 ul 2× sample buffer (250 mM Tris–HCL, 8% SDS, 40% glycerol, 0.04% bromophenol blue and 400 mM dithiothreitol); the proteins pulled down were analyzed by immunoblotting.

### Chromatin immunoprecipitation (ChIP) and Re-ChIP assays

The chromatin immunoprecipitation (ChIP) assays were performed according to the manufacturer’s protocol (Upstate Biotechnology, Inc., Lake Placid, NY, USA), with the exception of the conditions for sonication that were changed to five times for 30 s each at 10% output. The antibodies used for the immunoprecipitations were anti-ERα (HC-20X) (Santa Cruz Biotechnology, Inc), anti-hist1H2AC (Novus Biologicals), anti-LSD1 (ab17721, Abcam), anti-p300 CT (05-257) (Millipore), anti-RNA Pol α (05-623B) (Millipore) and anti-H3K9me2 (07-212) (Millipore). Nonspecific rabbit polyclonal antibodies were used as a negative control. After reversing the protein–DNA cross-linking of the immunoprecipitated complexes, DNA was extracted for real-time qPCR analysis. The primer sets used for the qPCR experiments are listed in Supplementary Table S2, presented as part of the Supplementary Material. For the Re-ChIP experiments, the complexes were eluted by incubating in 25 μl of 10 mM DTT for 30 min at 37°C. After centrifugation, the supernatant was diluted 20 times with Re-ChIP buffer and once again subjected to the ChIP procedure. Cross-linking was reversed by an overnight incubation at 65°C.

### 3C and ChIP-3C assays

We performed 3C assays (16) with some modifications. MCF-7 cells were fixed by incubating with 2% formaldehyde for 10 min at room temperature and quenched with 0.125 M glycine. After centrifugation for 15 min at 3500 rpm, the cells were suspended in lysis buffer (10 mM Tris–HCl, pH 8.0, 10 mM NaCl, 0.2% Nonidet P-40 and 1:500 complete protease inhibitor cocktail; Roche) for 90 min on ice. Following this, the nuclei were pelleted by centrifugation for 15 min at 2500 rpm, resuspended in 500 μl of 1× NEB buffer 4 containing 0.3% SDS and incubated at 37°C for 1 h. After adding Triton X-100 to a final concentration of 1.8% to sequester the SDS, the mixture was incubated at 37°C for 1 h. Following this, 800 U of BglII or NsiI was added, and the mixture was incubated at 37°C overnight to digest chromatin. The reaction was terminated by adding SDS to a final volume of 1.6%, and the mixture was heated to 65°C for 20 min. Ligation of DNA was carried out *in situ* for 4 h at 16°C using 0.5–2.0 ng/μl of chromatin in 800 μl of ligation buffer (NEB) containing 1% Triton X-100, and 30 Weiss Units of T4 ligase (NEB). After reversing the cross-linking by digesting with proteinase K at 65°C overnight, the DNA was purified by phenol-chloroform extraction followed by ethanol precipitation. The products of ligation were analyzed by PCR using primers located near BglII or NsiI cutting sites. The PCR products were gel-purified, cloned and sequenced. The primer sets used for 3C assays are listed in Supplementary Table S3, presented as part of the Supplementary Material.

The 3C/ChIP-loop assays were performed as described previously (17) with minor modifications. Briefly, immunoprecipitated chromatin was obtained as described earlier. Chromatin bound to the Dynabeads was digested with *Bgl*II or *Nsi*I, ligated with T4 DNA ligase (Promega), eluted and the cross-linking reversed. After purification, the ChIP-3C fragments were analyzed to detect short-range interactions using a set of primers targeting the ERα binding region and 3′-UTR site.

### Statistical analysis

All experiments were repeated two to five times, and the data were expressed as average ± SD. Statistical analysis of the data was conducted by pairwise Student's *t*-test. Differences were considered statistically significant (*) if *P* ≤ 0.05, and statistically very significant (**) if *P* ≤ 0.01.

## RESULTS

### H2ac is overexpressed in MCF-7 cells and is associated with ER+ breast cancer

**Figure 1.**
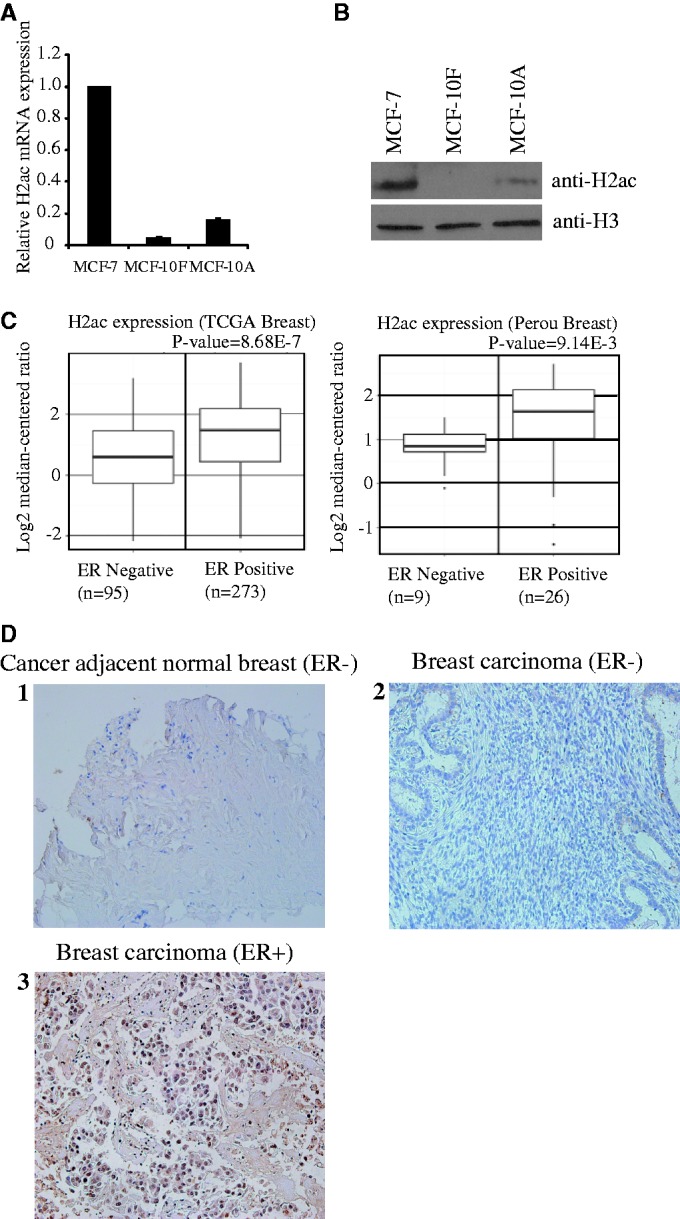
H2ac is overexpressed in MCF-7 cells and is associated with estrogen-receptor positive breast carcinoma. (**A**) mRNA expression levels of *H2ac* were determined by quantitative RT-PCR and normalized against 18s rRNA. (**B**) Western blotting of histone extracts prepared from MCF-7, MCF-10F and MCF-10A cells using the antibodies shown in the right panel. (**C**) Increased expression of H2ac mRNA in ER+ breast carcinomas from two independent data sets for human breast cancers. Data and statistics were obtained from the Oncomine database (14,15). (**D**) IHC analysis show H2ac protein expression in carcinoma samples of ER+ breast cancer patients. IHC was used to examine H2ac expression in TMAs and compare it with that of human ER− adjacent normal breast tissues, human malignant ER− breast cancer tissues and human malignant ER+ breast cancer tissues. Counterstaining used hematoxylin. Representatives of normal adjacent breast tissue (1), ER− malignant breast tissue (2) and ER+ malignant breast tissue (3) are shown.

During our investigation of the epigenetic regulation of gene expression in MCF-7 cells, we noticed that an H2A histone isotype, H2ac, was overexpressed in MCF-7 cells compared with non-tumorigenic MCF-10F cells (13). Subsequently, we performed real-time qPCR and immunoblotting to confirm the results of microarray analysis (Figure 1A). Our results confirmed that, at the protein level, H2ac is overexpressed in MCF-7 cells compared with MCF-10F and MCF-10A cells (Figure 1B). Analysis of H2ac mRNA expression also showed that this gene is highly upregulated in tumors (Supplementary Figure S1). Because MCF-7 cells are ERα+ breast cancer cells, we examined whether H2ac is overexpressed in ER+ breast cancers. We first performed Oncomine bioinformatic analyses of TCGA data sets (https://tcga-data.nci. nih.gov/) and found that *H2ac* mRNA expression was more closely associated with ER+ clinical breast cancer samples than with ER− samples (Figure 1C); (P = 8.68E-7 and 9.14E-3). To further examine the correlation between H2ac and ER expression in human ER+ breast cancer, we performed immunohistochemistry (IHC) on a tissue microarray panel (TMA) that included 122 breast cancer cases (34 ER+ breast carcinoma, 82 ER− breast carcinoma and 6 ER− cancer-adjacent normal breast tissues]. As shown in Figure 1D, H2ac protein was abundantly expressed in the analyzed ER+ breast cancer samples (85% of the cases, *P* < 0.001), whereas H2ac was observed in 24% of ER− breast cancer samples (*P* < 0.001) and 0% of ER− cancer-adjacent normal breast tissues. These results indicated that H2ac expression is associated with breast cancer, and particularly with ER+ breast cancer.

### H2ac is required for estrogen-stimulated cell proliferation and cell cycle progression

**Figure 2.**
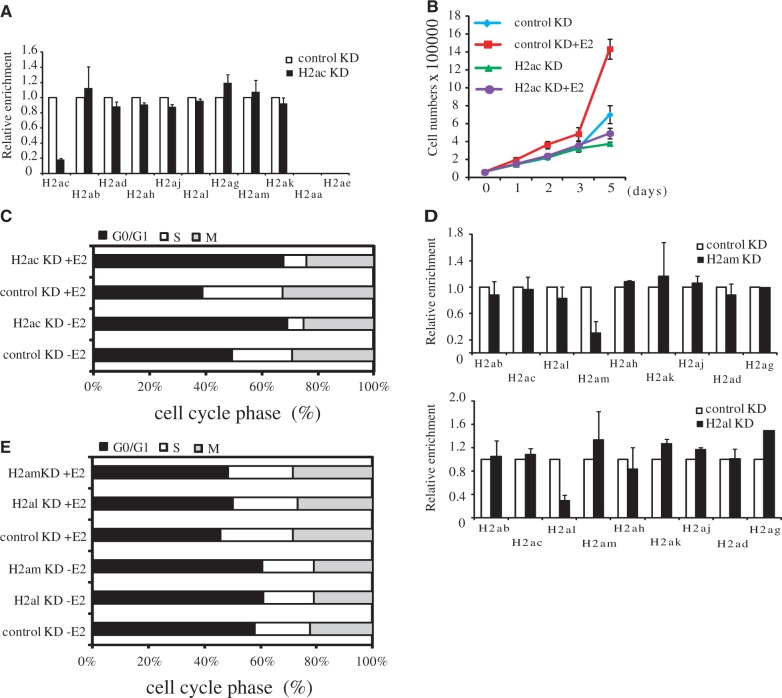
The effect of H2ac knockdown on cell proliferation and the cell cycle in E2-induced MCF-7 cells. (**A**) Expression of H2A isotypes was determined by RT-qPCR in H2ac-depleted MCF-7 cells and normalized against 18s rRNA. (**B**) Cell proliferation assays of depleted-H2ac MCF7 cells were performed using control siRNA or siH2ac in the absence (−E2) or presence (+E2) of estradiol. (**C**) Cell cycle profile analysis using propidium iodide (PI) staining and flow cytometry of MCF7 cells treated with control siRNA and siH2ac in the presence of either vehicle (−E2) or estradiol (+E2) for 24 h. (**D**) Expression of H2A isotypes was determined by RT-qPCR in H2al or H2am-depleted MCF-7 cells and normalized against 18s rRNA. (**E**) Cell cycle profile analysis using propidium iodide (PI) staining and flow cytometry of MCF7 cells treated with control siRNA, siH2al and siH2am in the presence of either vehicle (−E2) or estradiol (+E2) for 24 h.

Because H2ac was overexpressed in ERα+ MCF-7, where estradiol (E2) induces cell proliferation by binding to ERα, we investigated the role of H2ac in E2-dependent cellular proliferation. First, we performed siRNA-mediated knockdown of H2ac and analyzed cell proliferation by counting cell numbers; we also examined cell cycle distribution in E2-stimulated MCF-7 cells by fluorescence-activated cell sorting. To confirm the specific knockdown of H2ac, we analyzed the expression of H2A isotypes in H2ac-depleted MCF-7 cells. Our results showed that other H2A isotypes were not affected by H2ac depletion (Figure 2A). We also confirmed reduced H2ac protein expression in H2ac-knockdown cells by western blotting. Canonical H2A proteins were not affected by H2ac depletion in these cells (Supplementary Figure S2).

We found that H2ac knockdown had an impact on the E2-stimulated proliferation MCF-7 cells. Depletion of H2ac reduced the rates of E2-stimulated MCF-7 cell proliferation to a level comparable with that observed in the absence of E2 (Figure 2B). Consistent with this observation, flow cytometric analysis showed that knockdown of H2ac resulted in cell cycle arrest at the G1 phase in E2-stimulated MCF-7 cells (Figure 2C). To confirm that the specific depletion of H2ac led to cell cycle arrest, we transfected MCF-7 cells with siRNAs targeting either H2al or H2am, and performed flow cytometric analysis of the E2-stimulated transfected cells. The specificity of the H2al and H2am knockdowns were confirmed by real-time qPCR (Figure 2D). As shown in Figure 2E, neither the knockdown of H2al nor the depletion of H2am had an effect on cell cycle progression. These findings suggested that the specific depletion of H2ac led to loss of E2-stimulated cell proliferation and cell cycle progression.

### H2ac is required for the induction of estrogen-stimulated genes

**Figure 3.**
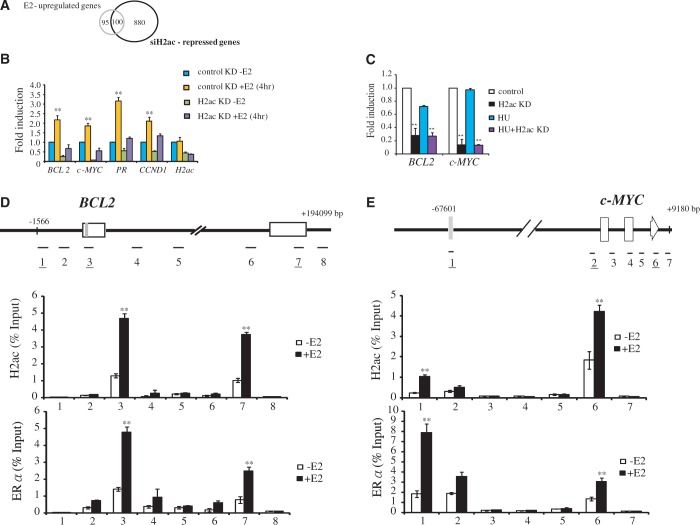
H2ac regulates E2-dependent gene transcription in MCF-7 cells. (**A**) A Venn diagram showing the number of E2 upregulated genes affected by knockdown of H2ac in MCF-7 cells. (**B**) Expression levels of *BCL2*, *c-MYC*, *CCND1*, *PR* and *H2ac* in MCF-7 cells depleted of H2ac using specific siRNA in the absence (−E2) or presence (+E2) of estradiol for 4 h. mRNA expression levels were determined by quantitative RT-PCR and normalized against 18s rRNA (**P < 0.01, *t*-test). (**C**) Expression levels of *BCL2* and *c-MYC* in HU-treated and H2ac-depleted MCF-7 cells. mRNA expression levels were determined by quantitative RT-PCR and normalized against 18s rRNA (**P < 0.01, *t*-test). (**D-E**) Upper panel: Schematic diagram of *BCL2* and *c-MYC* genes showing enhancers (gray bars), exons (open boxes) and primer sets (eight segments in the *BCL2* gene and seven segments in the *c-MYC* gene) used to produce the ChIP amplicons. The numbers of primer pairs covering enhancer, promoter and 3′UTR regions were underlined. Lower panel: Distribution of H2ac and ERα, respectively, in the *BCL2* and *c-MYC* gene. The specific antibodies used in the ChIP experiments were anti-H2ac and anti-ERα. DNA isolated from immunoprecipitated chromatin was subjected to qPCR to amplify the DNA fragments. Data represents mean ± SD for at least two independent experiments (** *P* < 0.01, *t*-test).

To elucidate the effect of H2ac knockdown on the expression of genes regulating cell proliferation and the cell cycle in E2-stimulated MCF-7 cells, we performed transcriptome analysis using microarray. We observed that 191 genes were upregulated >2-fold and 1734 genes were downregulated 72 h after H2ac knockdown. Notably, 100 of the 195 known genes upregulated by E2 (51%) were downregulated by the depletion of H2ac (Figure 3A). We then confirmed the results of microarray analysis for four ERα target genes, namely *BCL2*, *c-MYC*, *PR* and *CCND1*. As shown in Figure 3B, depletion of H2ac significantly suppressed E2-dependent induction of *BCL2*, *c-MYC*, *PR* and *CCND1* to an extent that their expression levels were similar to those observed in cells grown in estrogen-free medium. To examine whether the changes in expression of *BCL2* and *c-MYC* were due to cell cycle arrest, we performed real-time qPCR and analyzed the expression of *BCL2* as well as *c-MYC* in MCF-7 cells arrested at the G1 phase by treatment with hydroxyurea (HU). Our results showed that the expression of *BCL2* and *c-MYC* remained unchanged even in cell cycle arrested (G1) cells (Figure 3C), clearly suggesting that the downregulation of these genes did not occur as a result of cell cycle arrest.

To investigate the mechanism by which ERα target genes are regulated, we examined whether H2ac protein is associated with the *BCL2* and *c-MYC* genes by ChIP assay using an antibody directed against H2ac. Further, to determine the transcriptional regulation of *BCL2* and *c-MYC* following ERα activation, ChIP assays were performed at 0 and 30 min after stimulating MCF-7 cells with E2. We divided *BCL2* and *c-MYC* genes and their surrounding sequences into 8 and 7 segments, respectively, and performed ChIP experiments to analyze the presence of H2ac and ERα in these regions in the absence or the presence of E2. As expected, ERα was identified as binding to the ERα binding sites in the enhancer region of *BCL2* and in the distal 67-kb enhancer of *c-MYC* (Figure 3C and D) in E2-stimulated cells (18–20). Unexpectedly, ERα was also found to bind to the 3′-UTR of both genes. The ChIP analysis also revealed that H2ac co-localized with the ERα, bound to the various *BCL2* and *c-MYC* sequences (Figure 3C and D).

To demonstrate the specificity of gene expression regulation by H2ac, we tested two ER-induced genes, *TGIF2* and *PRKCE*, which the microarray data suggested were not affected by H2ac knockdown. Quantitative PCR analysis confirmed that the expression of these two ER-induced genes was not affected by H2ac depletion. Further, ChIP analysis showed that H2ac was not recruited to the promoters or the 3′-UTR regions of these two genes in E2-stimulated MCF-7 cells (Supplementary Figure S3A and B). These results suggested that H2ac was able to bind to and regulate only a subset of ERα-target genes.

### H2ac is required for ERα recruitment and its co-localization with the *BCL2* and *c-MYC* genes

**Figure 4.**
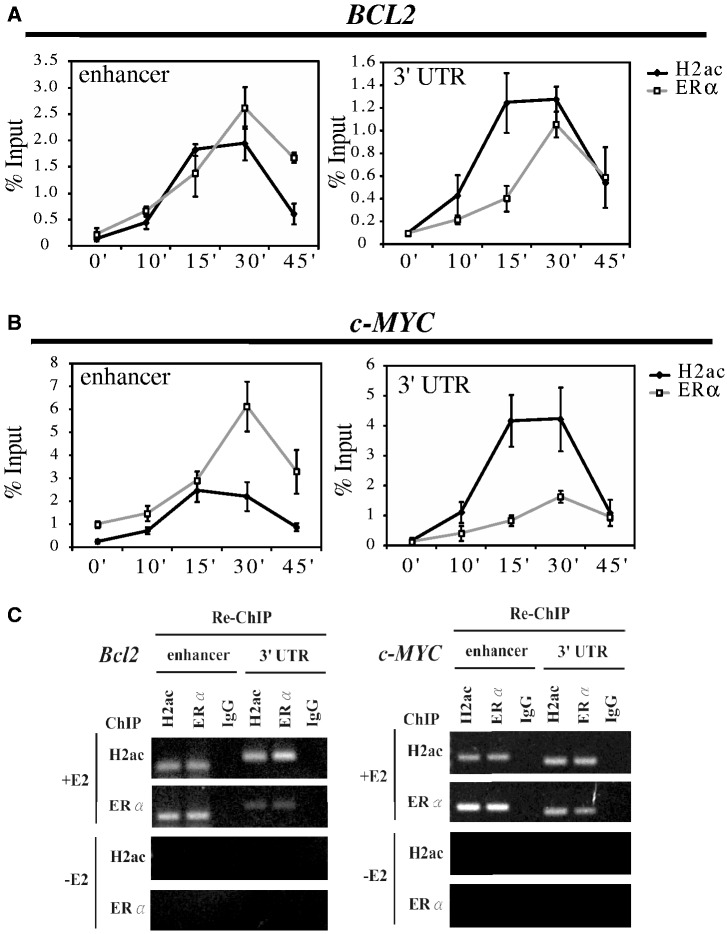
Recruitment kinetics of H2ac and ERα whereby H2ac co-localizes with ERα at the *BCL2* and *c-MYC* loci after E2 treatment of MCF-7 cells. (**A, B**) Kinetic ChIP experiments were performed using H2ac and ERα specified antibodies. After 2 h treatment with 2.5 μM α-amanitin, the cells were washed and placed in medium supplemented with 2.5% dextran/charcoal-treated FBS that included 10 nM estradiol (E2) to carry out the kinetic ChIP assay of H2ac and ERα. All ChIP were performed using a single chromatin preparation for each time point. (**C**) Re-ChIP assay of H2ac and ERα recruited to the promoter and 3′UTR regions of *BCL2* and *c-MYC*. Chromatin was prepared from MCF-7 cells treated for 30 min with 10 nM estradiol (E2) and this was then subjected to the ChIP procedure using the antibodies shown on the left side; the second immunoprecipitation was carried out using the antibodies shown at the top of the image.

To establish the order in which H2ac and ERα were recruited to the enhancers and 3′-UTRs of the *BCL2* and *c-MYC* genes, we performed ChIP analysis at 0, 10, 15, 30 and 45 min following E2 stimulation. These ChIP time course experiments, carried out after E2 treatment, revealed that the H2ac and ERα proteins were recruited rapidly to the enhancers and 3′-UTRs of the *BCL2* and *c-MYC* genes (Figure 4A and B). Additionally, a weakened H2ac association with *BCL2* and *c-MYC* observed 45 min after E2 treatment was found to correlate with attenuated ERα binding. To examine whether ERα and H2ac were present within the same multi-protein complex at various sites of these genes, we stimulated the cells with E2 for 45 min and performed ChIP/Re-ChIP experiments; this was accomplished by performing a first immunoprecipitation using H2ac antibody, followed by a second immunoprecipitation with ERα antibody. This method isolated DNA–protein complexes containing both ERα and H2ac. As shown in Figure 4C, H2ac and ERα were found to be present together at the ERα binding sites and at the 3′-UTRs of *BCL2* and *c-MYC* genes in an E2-dependent manner.

**Figure 5.**
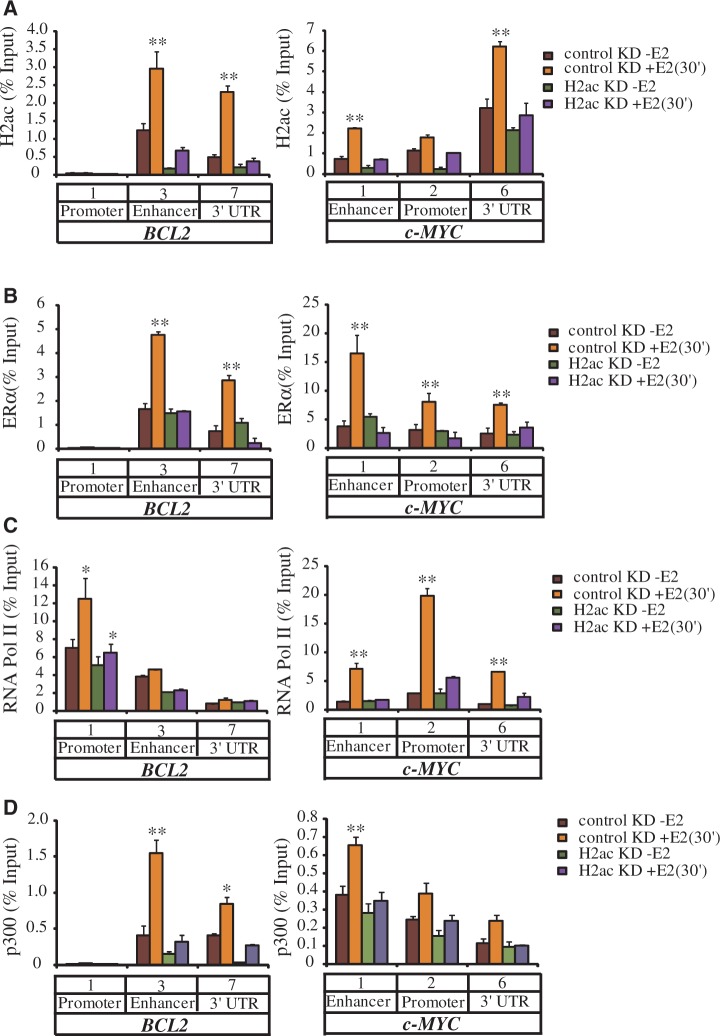
H2ac is required for the recruitment of ERα. (**A–D**) ChIP assays showing the effect of H2ac depletion on the occupancy of H2ac, ERα, p300 and RNA Pol II at the *BCL2* and *c-MYC* genes in the absence (−E2) or presence (+E2) of estradiol for 30 min. Data represent mean ± SD of at least three independent experiments (*P < 0.05; **P < 0.01, *t*-test).

To clarify whether it was H2ac that recruited ERα to the binding sites or vice versa, we analyzed ER binding to the target sites following depletion of H2ac (Figure 5A). Depletion of H2ac abolished recruitment of ERα as well as that of RNA polymerase II (RNA Pol II) to *BCL2* and *c-MYC* chromatin (Figure 5B and C) in E2-treated cells. It has been shown that ERα is acetylated by p300 acetylase to enhance ligand-dependent activity and p300 co-localization at ERα-binding regions (21,22). We examined whether H2ac was required for the recruitment of p300 to the ERα binding sites. Depletion of H2ac abolished the recruitment of both p300 and ERα to the *BCL2* and *c-MYC* chromatin in E2-treated cells (Figure 5D). These findings suggested that H2ac is essential for the recruitment of ERα and its cofactor, p300, to their target genes, and that this association is needed for the transcriptional regulation of a number of ERα target genes.

### H2ac forms a protein complex with ERα and controls the expression of various E2-induced genes

**Figure 6.**
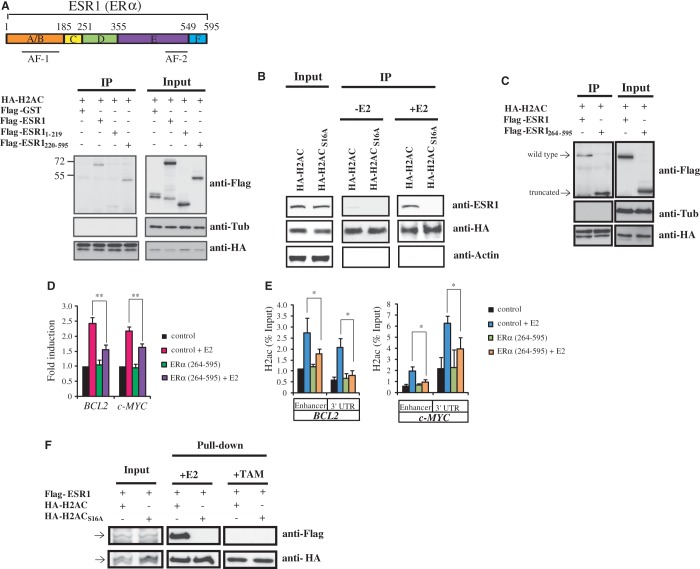
H2ac forms a complex with ERα to control the *BCL2* and *c-MYC* genes. (**A**) Schematic representation of the ERα functional domains. Immunoprecipitation analysis of the functional domains of ERα that interact with H2ac. Interactions between an *in vivo* transcribed and translated H2ac HA construct and ERα (ESR1) fragment Flag constructs were assayed by immunoblotting. (**B**) Co-IP analysis of how H2ac or the H2ac mutant interacts with endogenous ERα in the presence or absence of E2. (**C**) Co-IP analysis of how H2ac interacts with the truncated ERα_(264-595)_ that lacks a DNA binding domain. (**D**) Quantitative RT-PCR of the *BCL2* and *c-MYC* genes in control MCF-7 cells and in MCF-7 cells that are overexpressing the truncated ERα in the absence (−E2) or presence (+E2) of estradiol. The relative expression levels normalized against 18s rRNA are displayed (*n* = 3, mean ± S.D.) (**P < 0.01, *t*-test). (**E**) ChIP assays showing the effect of the truncated ERα on the occupancy of H2ac at the *BCL2* and *c-MYC* genes in the absence (−E2) or presence (+E2) of estradiol in MCF-7 cells. Data represent mean ± SD (*P < 0.05, *t*-test). (**F**) *In vitro* pull-down analysis of how ERα interacts with wild type or mutant of H2ac in the presence of E2 or TAM.

We investigated whether H2ac binds directly to ERα. To this end, we transfected MCF-7 cells with HA-tagged H2ac and FLAG-tagged ERα, and examined their association by performing immunoprecipitation followed by immunoblotting. Our results confirmed the binding of H2ac to ERα (Figure 6A). Additionally, HA-H2ac directly interacted and formed a complex with endogenous ERα in the E2-treated cells (Figure 6B). To determine whether the interaction between H2ac and ERα depended on the binding of ERα to DNA, we performed co-IP experiments using a truncated ERα that lacked the DNA binding domain. Our results showed that H2ac was able to associate with the truncated form of ERα lacking the DNA-binding domain (Figure 6C). Furthermore, overexpression of the truncated form of ERα interfered with the H2ac-mediated induction of *BCL2* and *C-MYC* gene expression in E2-treated MCF-7 cells (Figure 6D). ChIP assays also revealed that, in E2-treated MCF-7 cells overexpressing the truncated form of ERα lacking the DNA binding domain, the levels of H2ac in *BCL2* and *c-MYC* chromatin were significantly lower (Figure 6E). These findings suggested that the DNA-binding domain of ERα is important for the binding of ERα–H2ac complex to the genes.

To demonstrate that ERα and H2ac formed a complex in the absence of DNA, we synthesized the proteins by *in vitro* transcription–translation and analyzed their E2-induced association by pull-down assay. Our results unambiguously established that H2ac directly interacted with ERα in the absence of DNA (Figure 6F). Taken together, these results clearly suggested that H2a formed a complex with ERα in E2-treated MCF-7 cells, and that this complex binds to a series of target sites involved in regulating gene expression.

### The H2ac–ERα complex controls the transcription of various estrogen-induced genes by forcing the formation of a chromatin loop between regulatory sites

**Figure 7.**
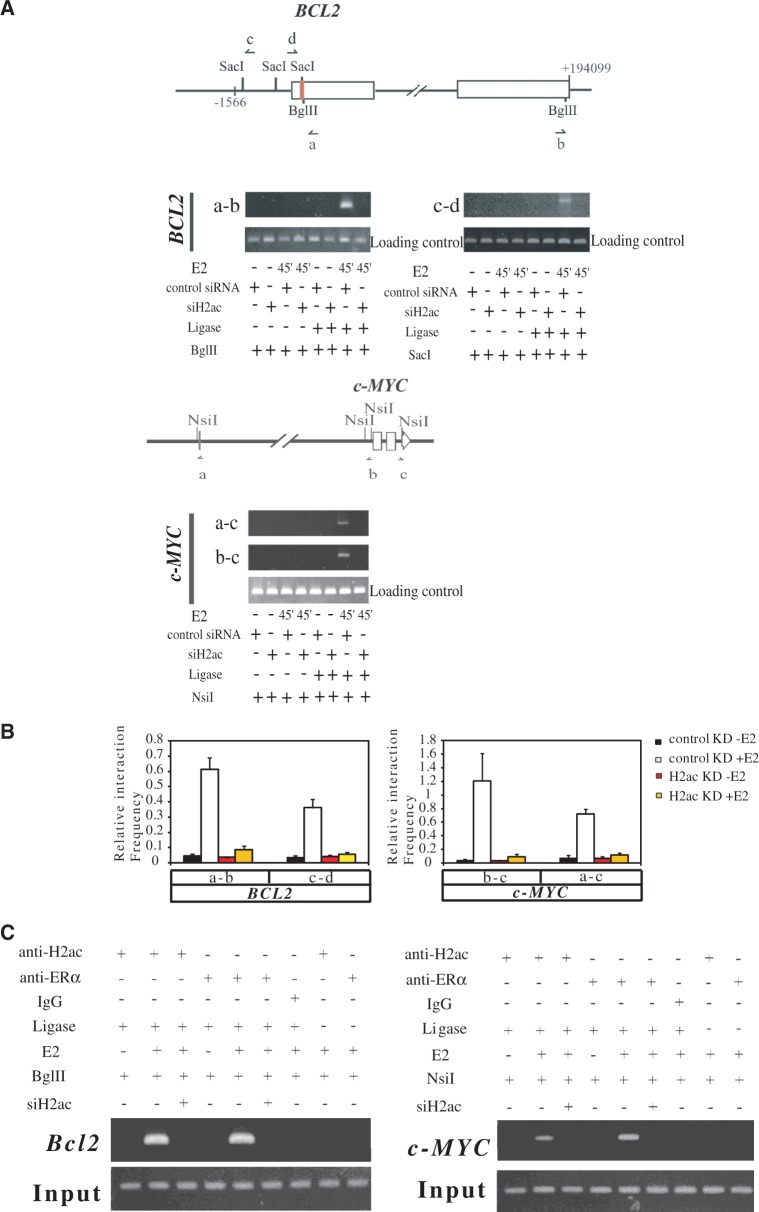
H2ac is required for the recruitment of ERα via loop formation. (**A**) Upper panel: Schematic diagram of the *BCL2* and c-MYC genes showing the coding region (open boxes), and enhancers (red bars).The restriction sites and the orientation of the 3C primers (arrows) for PCRs in 3C experiments are shown. Lower panel: 3C assays showing the presence in MCF-7 cells of the specific 3C products from the *BCL2* gene and from the *c-MYC* gene under conditions with or without H2ac knockdown. The experiments involved formaldehyde (CH_2_O) cross-linking and ligation of the samples in absence or presence of estradiol for 45 min. The loading control primers are shown in Supplementary Table S3. (**B**) Quantitative histograms of the 3C assays. Relative cross-linking frequencies are shown for the different ligation products that were quantified using primer pairs targeting the promoters, enhancers and 3′UTRs of *BCL2* and *c-MYC*. These were compared with a control primer pair and carried out under conditions with or without H2ac knockdown in absence or presence of estradiol for 45 min. Data represent mean ± SD of at least three independent experiments. (**C**) ChIP-3C analysis of the enhancer-3′UTR association of *BCL2* and of *c-MYC*. ChIP-3C assays were performed using H2ac and ERα specified antibodies to immunoprecipitate cross-linked chromatin from MCF-7 cells treated with E2 or either vehicle for 45 min; the products were then digested with Bgl II or Nsi I, followed by a ligation reaction in the presence or absence of DNA ligase. a–b primer pair for *BCL2* and a–c primer pair for *c-MYC*. The enhancer region of BCL2 was used as an input control.

Because H2ac and ERα demonstrated binding activity to both the enhancer region and the 3′-UTR of regulated genes, we hypothesized that the two regulatory elements may interact via a chromatin loop formed through protein–protein association. An analysis of the *in vivo* chromatin conformation of these regions using the chromatin conformation capture technique (16) confirmed this expectation (Figure 7A and B). Further, knockdown of H2ac abolished the association between the enhancer, promoter and 3′-UTR of *BCL2* and *c-MYC*, an observation that supported the hypothesis that the chromatin looping of these genes is dependent on the presence of H2ac. To further understand the role of H2ac in the formation of a chromatin loop, we performed a ChIP-3C assay using extracts from control and E2-treated MCF-7 cells. Products of ligation containing the ERα binding site and 3′-UTR could be detected in E2-stimulated cells, but not in vehicle-treated and H2ac-depleted MCF-7 cells. This supported the possibility that H2ac mediates association of chromatin between the ERα binding sites and 3′-UTRs of *BCL2* and *c-MYC* (Figure 7C), likely by forcing the formation of a double chromatin loop.

**Figure 8.**
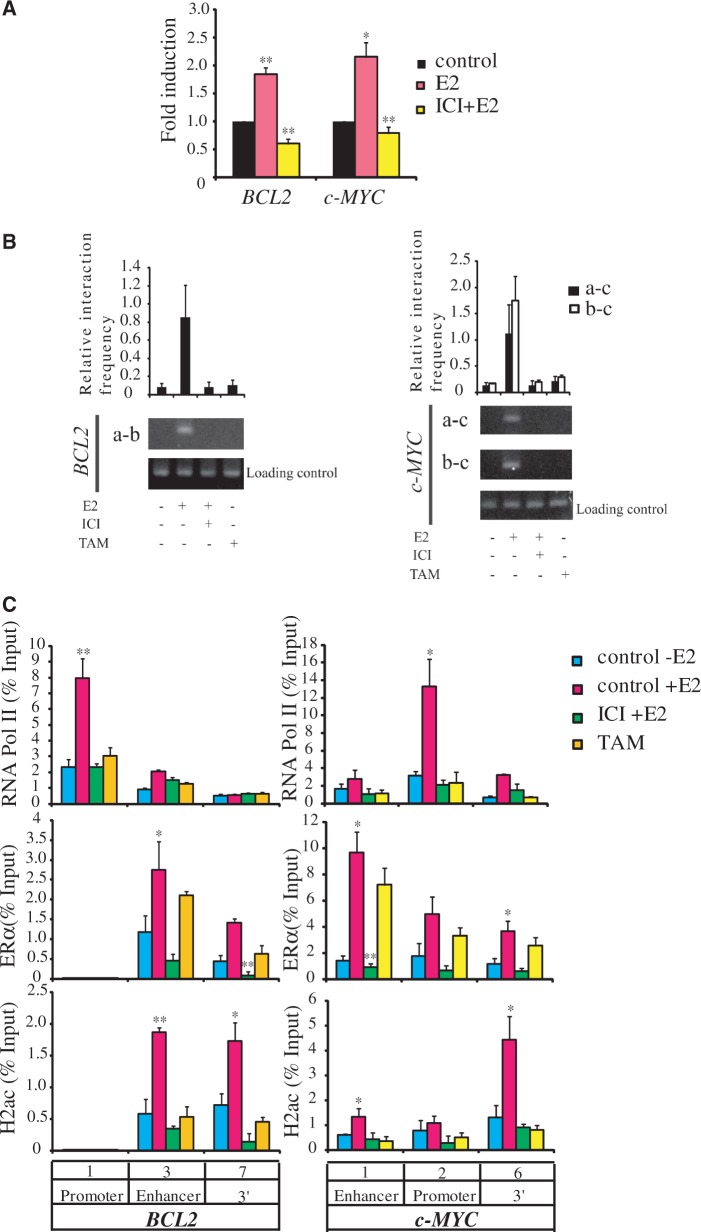
H2ac and ERα cooperate to regulate E2-dependent genes by loop formation. (**A**) Expression levels of *BCL2* and *c-MYC* in MCF7 cells that has undergone inhibition of ERα by ICI, and carried out in the absence (−E2) or presence (+E2) of estradiol for 4 h. mRNA expression levels were determined by qRT-PCR and normalized against the expression level of 18s rRNA (*P < 0.05; **P < 0.01, *t*-test). (**B**) Quantitative histogram and gel panels of 3C assays showing the presence of the specific 3C products from the *BCL2* and *c-MYC* genes with or without ICI or TAM treatment. (**C**) ChIP analysis of RNA Pol II, ERα and H2ac recruitment before and after treatment with E2 or TAM for 30 min at the *BCL2* and *c-MYC* loci of MCF-7 cells (*P < 0.05; **P < 0.01, *t*-test).

Treatment of cells with ICI 182580 leads to rapid degradation of ERα by the ubiquitin/proteasome pathway (23,24). To determine the role of ERα in chromatin looping, we stimulated cells with E2 following incubation with ICI 182580 and performed 3C assays to detect the interaction between the 3′-UTRs and enhancers of *BCL2* as well as *c-MYC*. Treatment of cells with ICI 182580 downregulated both *BCL2* and *c-MYC* expression (Figure 8A) and inhibited chromatin loop formation (Figure 8B). Similar results were obtained using TAM, which acts by competing with estradiol (E2) for binding to ERα. TAM binding is known to induce conformational changes in ERα that blocks the interaction of ERα with a number of coactivators, including AIB1, p300 and CBP (Figure 8B) (22). Our findings supported the hypothesis that ERα mediates association of chromatin between the ERα binding site and 3′-UTR (Figure 7C).

To clarify whether ERα was required for the E2-induced recruitment of H2ac to the *BCL2* and *c-MYC* chromatin, we examined the recruitment of H2ac and ERα to the enhancer and 3′-UTR regions following treatment with the ERα antagonists ICI 182580 or TAM. As expected, estradiol treatment induced recruitment of ERα together with H2ac to the enhancer regions of *BCL2* and *c-MYC*. Preincubation of MCF-7 cells with ICI 182580 abolished E2-induced recruitment of both ERα and H2ac. Further, ICI 182580 and TAM also interfered with the recruitment of RNA Polymerase II to the *BCL2* and *c-MYC* chromatin (Figure 8C). Because TAM binding affects the AF-2 domain of ERα, the above results suggested that H2ac binding to ERα depended on E2 signaling via the AF-2 domain. H2ac was found to bind to amino acids 220–595 and 264–595 of the C-terminal fragments of ERα containing the AF-2 domain, but not to the N-terminal fragment (Figure 6A and C). Results of the pull-down assay also showed that the presence of TAM abolished H2ac interaction with ERα (Figure 6F). These results indicated that the recruitment of ERα and H2ac to target genes was dependent on E2 signaling through AF-2 domain.

### The HAR domain of H2ac regulates ERα recruitment through H3K9 demethylation

**Figure 9.**
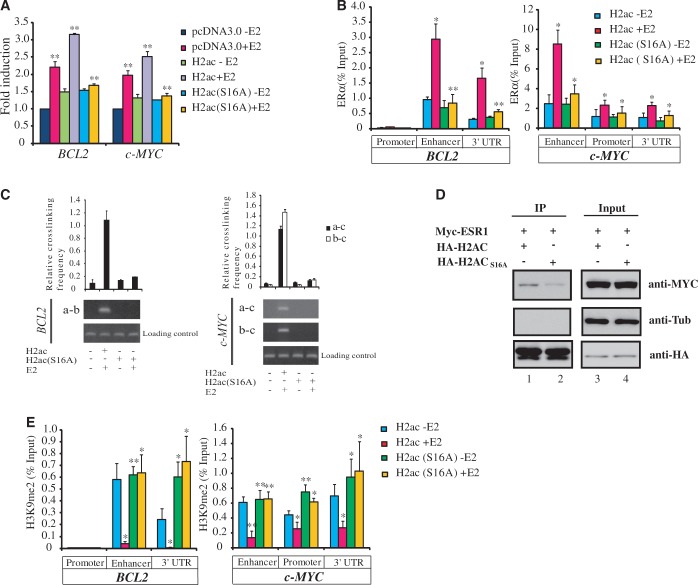
The HAR domain of H2ac regulates ERα recruitment via H3K9 demethylation. (**A**) Quantitative RT-PCR of the *BCL2* and *c-MYC* genes in control MCF-7 cells and in MCF-7 cells overexpressing mutant H2ac (S16A) in the absence (−E2) or presence (+E2) of estradiol. Relative expression levels normalized against 18s rRNA are displayed (*n* = 3, mean ± S.D.) (**P < 0.01, *t*-test). (**B**) ChIP assays showing the effect of the H2ac mutation on the occupancy in MCF-7 cells of ERα at the *BCL2* and *c-MYC* genes in the absence (−E2) or presence (+E2) of estradiol. Data represent mean ± SD for at least three independent experiments (*P < 0.05; **P < 0.01, *t*-test). (**C**) Quantitative histogram and gel panels showing 3C assays that indicate the presence in MCF-7 cells of the specific 3C products of the *BCL2* and *c-MYC* genes under conditions where there is wild type or mutant H2ac present; these experiments were carried out in the absence or presence of estradiol. (**D**) Diminished interaction of ERα with the mutant H2ac (S16A). Cells were transfected with different combinations of the pCMV-based expression plasmids. Whole-cell extracts from the transfected cells were immunoprecipitated (IP) with anti-HA agarose, and then analyzed by immunoblotting (lanes 1, 2); they were probed with anti-myc, anti-tubulin or anti-HA, antibodies as indicated on the right of the panels. In addition, 10% of the whole-cell extracts used for the individual IP reactions were also loaded as the ‘Input’ controls and probed with the same antibodies (lanes 3, 4). (**E**) ChIP analysis of histone H3K9me2 associated with the *BCL2* and *c-MYC* genes using MCF-7 cell overexpressing the H2A mutant. Data represent mean ± SD from at least three independent experiments (*P < 0.05; **P < 0.01, *t*-test).

Because there are 11 isotypes of H2A in the human genome, we wondered why H2ac, in particular, was involved in gene regulation through its interaction with ERα. Zheng *et al.* (25) reported that H2A in yeast contains a HAR domain that is involved in cross talk between histones H2A and H3 to regulate gene transcription. Examination of H2A isotypes showed that only H2ac and H2aa (Hist1H2AA) harbored this domain, and that H2aa is silenced in MCF-7 cells (Figure 2A). Therefore, we examined whether this domain was involved in the gene regulatory functions of H2ac. We mutated amino acid residue 16 in the HAR domain of H2ac, changing it from serine to alanine. Overexpression of this mutant in MCF-7 cells prevented the E2-induced upregulation of *BCL2* and *C-MYC* gene expression (Figure 9A). The mutant also prevented the recruitment of ERα to its target sites (Figure 9B) and inhibited the formation of chromatin loops (Figure 9C). Results of immunoprecipitation and *in vitro* pull-down assays also showed weak binding of this mutant to ERα as compared with the wild-type protein (Figure 6B, F and 9D). We also tested whether the other HAR domain mutants were abolished to interact with ERα. As shown in Supplementary Figure S4, all the HAR mutants of H2ac were abolished to interact with ERα.

Perillo *et al.* (26) showed that H3K9me2 is involved in ERα-mediated transcription. Since HAR domains have been shown to interact with H3, we examined whether H2ac mediated ERα-dependent changes in gene transcription via changes in the cellular levels of H3K9me2. We found that, in cells overexpressing wild-type H2ac, global levels of heterochromatic H3K9me2 was significantly reduced following E2 treatment, whereas these levels were not significantly affected in cells overexpressing the HAR domain mutant (Supplementary Figure S5). Specifically, ChIP assays revealed that, following E2 treatment, the levels of H3K9me2 in *BCL2* and *c-MYC* chromatin were significantly reduced in MCF-7 cells, whereas its levels were not significantly altered in cells overexpressing the HAR mutant (Figure 9E). These results suggested that H2ac activated transcription downstream of ERα activation by recruiting ERα to its target genes, as well as by suppressing H3K9me2 modification. Because LSD1 relieves histone repression by demethylation of histone H3 at lysine 9 (H3K9) (27,28), we examined whether LSD1 is involved in the H2ac-mediated regulation of gene expression. LSD1 knockdown led to attenuated *BCL2* and *c-MYC* expression in E2-stimulated cells (Supplementary Figure S6B). Further, ChIP analysis showed that, in E2-treated H2ac-overexpressing cells, LSD1 was recruited to ERα enhancer binding sites (Supplementary Figure S6A). Overexpression of H2ac HAR-domain mutant suppressed the binding of LSD1 at the ERα enhancer binding sites of E2-treated cells.

## DISCUSSION

In this report, we showed that H2ac acts as a novel mediator of transcription and is essential for the E2-dependent gene transcription and regulation of *BCL2* and *c-MYC* gene expression. At least in the case of *BCL2* and *c-MYC,* H2ac carries out this role by recruiting ERα to form activated chromatin loop structures. We further showed that H2ac directly interacts with ERα to modulate the transcription of ERα-regulated genes. This regulation of gene expression involves interaction between the gene's promoter, enhancer and 3′-UTR. Because depletion of H2ac leads to a severe defect in the E2-dependent signaling pathway and cell cycle arrest at G0/G1 phase, our findings identify H2ac as a novel mediator of tumorigenesis. Therefore, H2ac may be a useful target for developing new and improved therapeutics.

Estrogen signaling by ERα is a dynamic and complex process that requires the functioning of multiple coactivators, chromatin remodelers and corepressors during the phase of gene transcription (29,30). The dynamic assembly and dissolution of complexes following ERα activation and deactivation turns the expression of ERα target genes ‘on’ and ‘off’, respectively. The estrogen-induced rapid recruitment of ERα, H2A.Z, JMJD3 and p300 to the ERα binding sites of E2-dependent genes occurs within 30 min of the addition of estrogen (11,20,22,31). In our work, we have shown that H2ac rapidly co-localizes with ERα and p300 at the ERα binding sites of *BCL2* and *c-MYC* within 30 min of E2 treatment. Unexpectedly, H2ac and ERα were also found to bind to the 3′-UTRs of both genes. Recent genome-wide analysis of ERα binding sites have identified long-distance gene regulation by the ERα (19,32). These studies concluded that the majority of ERα binding sites are located >5 kb away from the transcriptional start site of E2-regulated genes (18,19,32), and that the regulation by ERα often involves interaction between multiple ER binding sites that are located far apart via formation of loops (33–35). Here, we showed that H2ac performs the critical task of recruiting ERα and p300 to the ERα binding sites, resulting in the formation of long-distance chromatin loops between various regulatory sites. Furthermore, we showed that the 3′-UTRs also participate in this process via the formation of double chromatin loops. Because treatment with the ER antagonists ICI or TAM also blocked the recruitment of H2ac to *BCL2* and *c-MYC* chromatin regions, we conclude that ERα and H2ac cooperate to critically regulate E2-dependent gene transcription. H2ac forms a complex with ERα through the AF-2 domain of ERα; this region is important for the recruitment of a number of regulatory factors, including p300, SRC-1 and GRIP1, to ERα target genes (22,36).

To study the role of the HAR domain of H2A in the modification of other histones and in gene transcription, Zheng *et al*. (25) constructed several mutants of the HAR domain of H2A from *Saccharomyces cerevisiae*. However, these mutants were screened only for histone modifications using bulk chromatin. Whether these HAR domain mutants affected transcription via the recruitment of regulatory factors remains unknown. Here, we showed that a mutation at serine 16 of the HAR domain of H2ac interfered with the transcriptional regulation of the ERα target genes, *BCL2* and *c-MYC*, by suppressing the interaction between ERα and H2ac. We also showed that cellular H3K9me2 levels affected H2ac-mediated ERα activation via the HAR domain. H3K9me2 is a repressive epigenetic modification that appears to be the most abundant heterochromatic modification, and has recently been reported to cover large domains in differentiated cells (27,37). Interestingly, overexpressing wild-type H2ac after E2-treatment reduced the global levels of H3K9me2, whereas the mutant form of HAR domain does not. Therefore, the HAR domain of H2ac influences methylation of H3K9 in E2-treated breast cancer cells. Perillo *et al*. (26) showed that demethylation of H3K9me2 is involved in ERα-mediated transcription, and that this process involved activation of the resident LSD1 histone demethylase. LSD1 regulates the activation of the *BCL2* gene via the formation of a chromatin loop structure between the enhancer and the proximal promoter regions (26). We found that the HAR domain mutant interfered with the recruitment of LSD1 to the enhancers of *BCL2* and *c-MYC*, and depletion of LSD1 resulted in the inactivation of both genes. Further, we showed that H2ac is associated with LSD1 *in vivo*. Taken together, our findings suggest that H2ac activates genes through the recruitment of the H3K9 demethylase, LSD1, and that the HAR domain of H2ac plays a key role in this process.

Past studies have shown that incorporation of histones into promoter nucleosome control gene expression in response to appropriate physiological signals (11,38,39). We demonstrated that H2ac interacts with the truncated ERα lacking the DNA binding domain, and that H2ac and ERα directly interacted in response to E2 stimulation. In our H2ac mutant experiment, immunoprecipitation analysis also showed that the binding of the mutant to ERα is greatly diminished. These results demonstrated that H2ac directly interacts with activated ERα in a DNA-independent manner. Further, truncated ERα lacking the DNA binding domain competed with wild-type ERα for binding to H2ac and interfered with E2-dependent gene activation. Compared with E2-stimulated MCF-7 cells, reduced levels of H2ac were found to be associated with *BCL2* and *c-MYC* chromatin in E2-treated cells overexpressing the truncated form of ERα. These results clearly suggested that the DNA-binding domain of ERα is important for binding of the ERα–H2a complex to various genes.

A recent report showed that H2ac was overexpressed in CD19+ B cells and bladder epithelial cells from healthy individuals when compared with cancer cells, and that knockdown of H2ac led to increased proliferation of 293TN and U2OS cells (40). In contrast, our results demonstrated that H2ac is overexpressed in ERα+ breast cancer samples, and that E2 upregulates *BCL2* mRNA expression via long-range communication between distal regulatory elements and proximal promoter regions through the E2-dependent formation of H2ac–ERα signaling complexes. Depletion of H2ac in MCF-7 cells suppressed the E2-dependent expression of *BCL2* and inhibited E2-stimulated cell proliferation. A number of genes involved in tumorigenesis have been shown to act as either oncogenes or tumor suppressor genes, depending on the cellular context (41). For instance, transforming growth factor beta-1 has been known to act, in a context-dependent manner (42), as an oncogene (43,44) or a tumor suppressor (45). It is likely that, H2ac may also play dual roles in tumorigenesis.

In conclusion, we demonstrated for the first time that H2ac, a histone that contains the HAR domain, acts as a master regulator of E2-dependent gene expression in ERα+ breast cancer cells. This histone cooperates with ERα to activate target genes by recruiting specific activators that mediate interaction between the promoter, the enhancer and the 3′-UTR of the target gene. To our knowledge, this is the first report of a housekeeping histone serving as a critical regulator of gene transcription. Our findings indicate the possibility that other histones may also be involved in the regulation of transcriptional programs in a cell type-specific and context-dependent manner. The upregulation of oncogenes by H2ac via the recruitment of activators is a new mechanism of tumorigenesis. This pathway may be a useful target for the discovery and development of new anticancer agents.

## SUPPLEMENTARY DATA

Supplementary Data are available at NAR Online.

## FUNDING

Funding for open access charge: Ministry of Education, Aim for the Top University Plan.

*Conflict of interest statement*. None declared.

## Supplementary Material

Supplementary Data
