# Supplementary material for: An H2A histone isotype regulates estrogen receptor target genes by mediating enhancer-promoter-3′-UTR interactions in breast cancer cells
Source: Nucleic Acids Res. 2013 Dec 25;42(5):3073–88. doi: 10.1093/nar/gkt1341 (PMC3950719; doi:10.1093/nar/gkt1341)
Supplement: Supplementary Data [file supp_42_5_3073__index.html]

An H2A histone isotype regulates estrogen receptor target genes by mediating enhancer-promoter-3′-UTR interactions in breast cancer cells — Supplementary Data 

# An H2A histone isotype regulates estrogen receptor target genes by mediating enhancer-promoter-3′-UTR interactions in breast cancer cells

## Supplementary Data

files

**Files in this Data Supplement:**

- Supplementary Data - pdf file
