# Supplementary material for: An H2A histone isotype regulates estrogen receptor target genes by mediating enhancer-promoter-3′-UTR interactions in breast cancer cells
Source: Nucleic Acids Res. 2013 Dec 25;42(5):3073–88. doi: 10.1093/nar/gkt1341 (PMC3950719; doi:10.1093/nar/gkt1341)
Supplement: Supplementary Data [file supp_gkt1341_nar-02481-v-2013-File011.pdf]

# Supplementary Figures

# Supplementary Figure S1

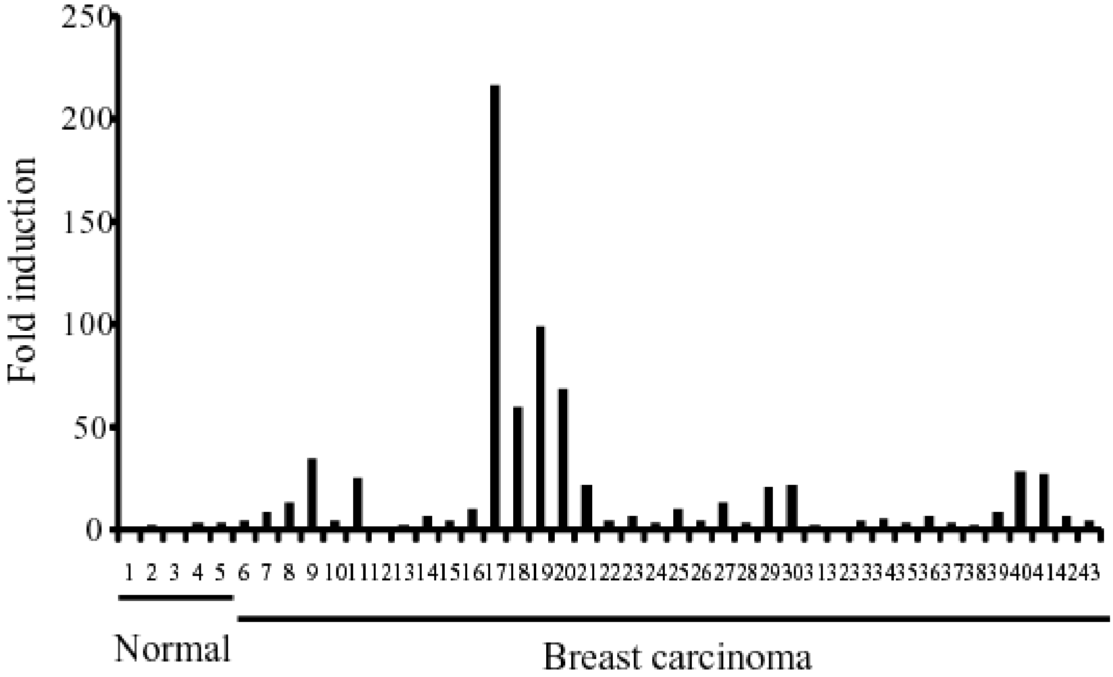


**Supplementary Figure S1.** Quantitative RT-PCR of *H2ac* in 38 breast carcinoma cDNA samples and 5 normal breast tissues. (Human Breast cancer panel samples from ORIGENE)

# Supplementary Figure S2

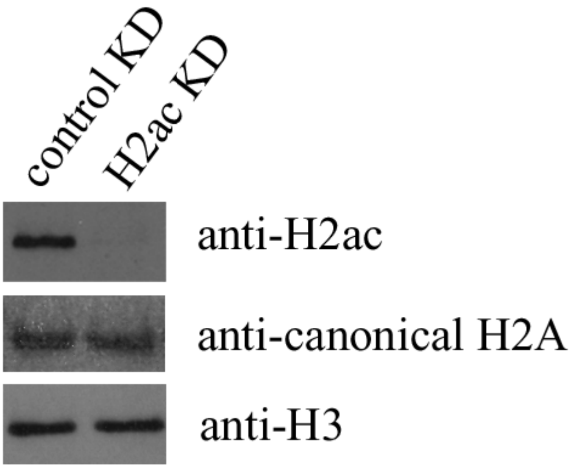


**Supplementary Figure S2.** Western blotting of histone extracts prepared from MCF-7 depleted of H2ac or control Knockdown cells using the antibodies shown in the right panel.

# Supplementary Figure S3

## A

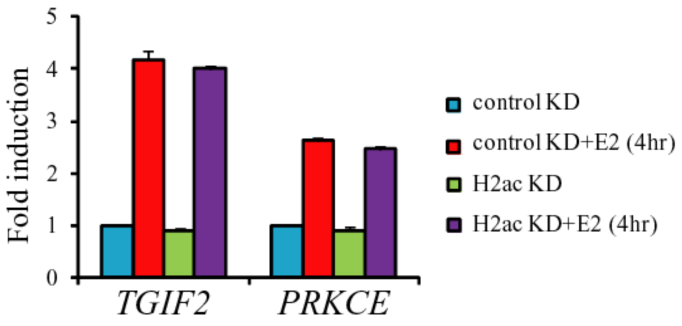


## B

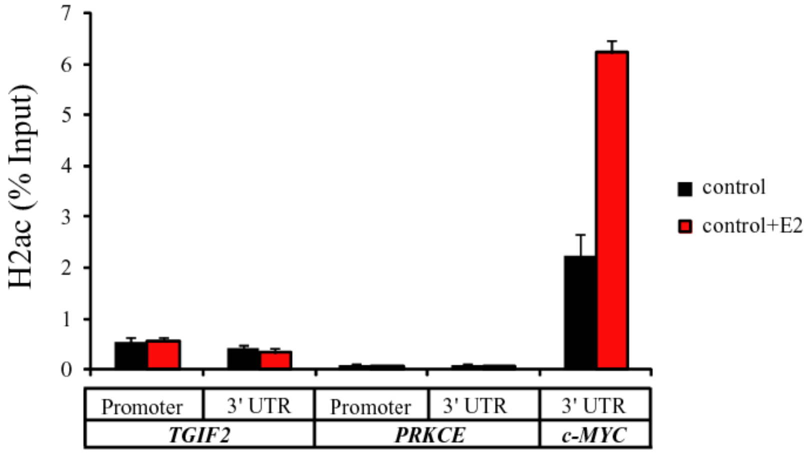


**Supplementary Figure S3.** *TGIF2* and *PRKCE* of E2-inducted genes in H2ac-independent manner. (**A**) Expression levels of *TGIF2* and *PRKCE* in MCF-7 cells depleted of H2ac using specific siRNA in the absence (-E2) or presence (+E2) of estradiol for 4 h. mRNA expression levels were determined by Quantitative RT-PCR and normalized against 18s rRNA (** $p < 0.01$, *t*-test). (**B**) ChIP assay showing distribution of H2ac in promoter and 3' UTR regions of *TGIF2* and *PRKCE* genes in the absence (-E2) or presence (+E2) of estradiol for 30 min. The 3' UTR region of *c-MYC* as positive control. Data represents mean±S.D.

.

# Supplementary Figure S4

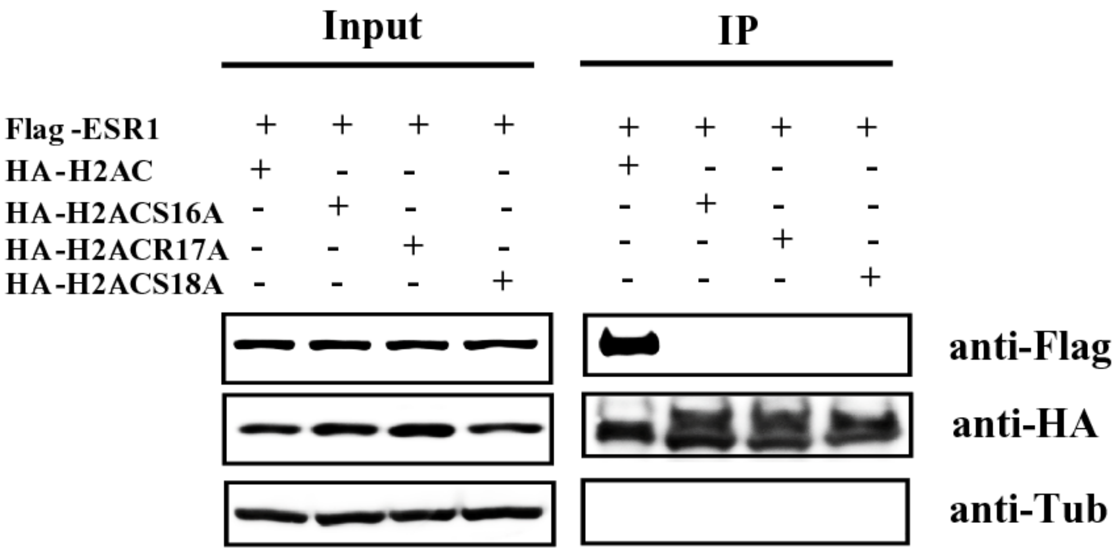


**Supplementary Figure S4.** Diminished interaction of Flag-ERα with the mutant H2AC (S16A, R17A and S18A). Whole-cell extracts from the transfected cells were immunoprecipitated (IP) with anti-HA agarose, and then analyzed by immunoblotting; they were probed with anti-flag, anti-tubulin, or anti-HA, antibodies as indicated on the right of the panels. In addition, 10% of the whole-cell extracts used for the individual IP reactions were also loaded as the “Input” controls and probed with the same antibodies.

# Supplementary Figure S5

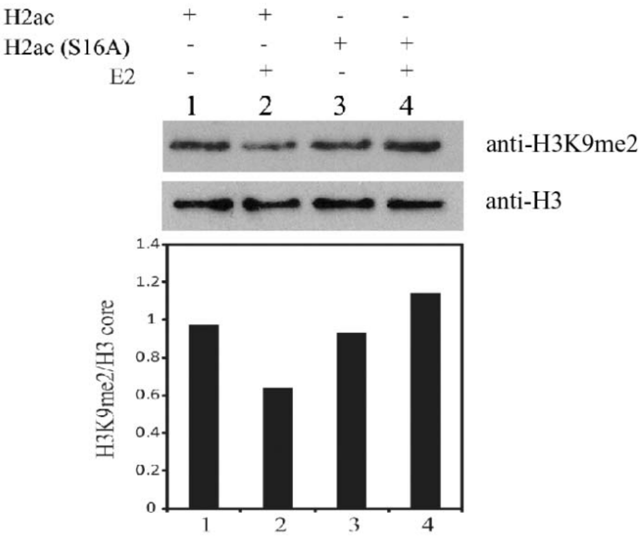


**Supplementary Figure S5.** Western blotting of histone extracts prepared from cells containing the wild-type and mutant proteins using the antibodies shown in the right panel. Histone modification levels were normalized against the amount of histone H3 present. (N=2)

# Supplementary Figure S6

## A

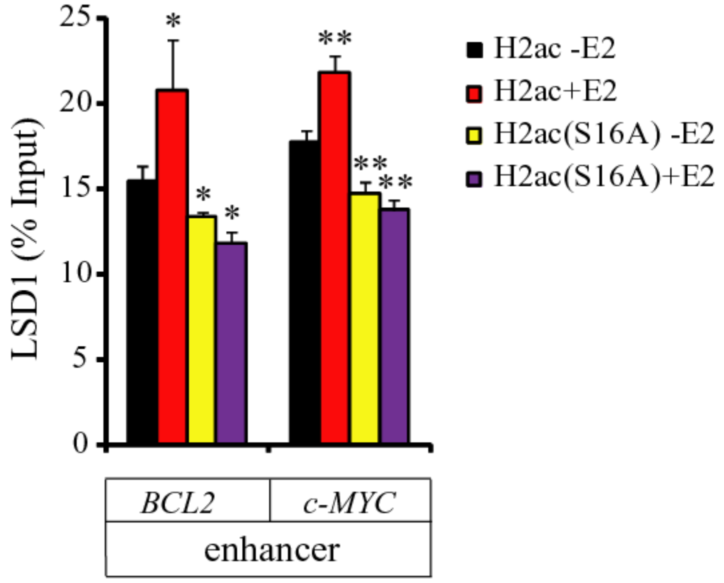


## B

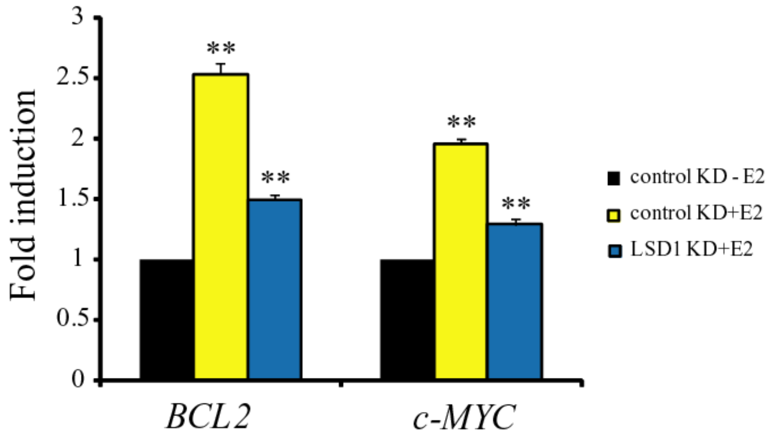


**Supplementary Figure S6.** Transcriptional regulation of E2-inducted genes by LSD1. (**A**) ChIP assay showing that LSD1 occupancy at the enhancer of *BCL2* and *c-MYC* in wild type H2ac or H2ac mutant cells in the absence (-E2) or presence (+E2) of estradiol (N = 3, mean ± S.D.) (* $p < 0.05$ ;** $p < 0.01$, *t*-test). (**B**) Quantitative RT-PCR of *BCL2 and c-MYC* genes in control siRNA treated and LSD1-depleted MCF-7 cells in the absence (-E2) or presence (+E2) of estradiol. A relative expression normalized over 18s rRNA is displayed (** $p < 0.01$, *t*-test).

# Supplementary Tables

**Supplementary Table S1.** Primers for qRT-PCR assay

| Gene | Sequences |
|---|---|
| *c-MYC* | 5' TCTTCCCCTACCCTCTCAACGA 3' |
| | 5' AGTGGGCTGTGAGGAGGTTTG 3' |
| *BCL2* | 5' TGTGTGGAGAGCGTCAACCG 3' |
| | 5' CTTTAGTGAACCTTTTGCATATTTGT 3' |
| *PR* | 5' GGGCAATGGAAGGGCAGCAC 3' |
| | 5' AACTCTGACTTTATTGAACTTTTT 3' |
| *CCND1* | 5' CCCGATGCCAACCTCCTCAA 3' |
| | 5' TCGCACTTCTGTTCCTCGCA 3' |
| *H2ac* | 5' CCGCTGGTTTTGGTGATTTTTGTC 3' |
| | 5' CGCCTGCCCCAACCCGCTCT 3' |
| 18s rRNA | 5'GCCCGAAGCGTTTACTTTGA 3' |
| | 5' TCCATTATTCCTAGCTGCGGTATC 3' |
| *RPS 13* | 5’ GGACTTGCTCCTGATCTTCCTG 3’ |
| | 5’ CTTATAATATCGAGCCAAACGGTG 3’ |
| H2ab | 5' CTGCCTAAGAAAACTGAGAGCC 3' |
| | 5' GCTGTTAGGCTGATTTTGTCTGC 3' |
| H2ad | 5' GCATCATCCCCCGACACCTG 3' |
| | 5' TCTCGTTTTACTTGCCCTTGG 3' |
| H2ah | 5' CCGTATCATCCCGCGTCACC 3' |
| | 5' TTCACAACCTCGCTCCTTATTT 3' |
| H2ak | 5' TGGACCGAGGTATGAGTAATGAAC 3' |
| | 5' GCTGTTAGGCTGATTTTGTCTGC 3' |

| | |
|---|---|
| H2aj | 5' CCGCGTCACCTCCAGCTGGC 3' |
| | 5' CGCTTTTCAACTCGGTCTTTACTTA 3' |
| H2aa | 5' ATTTGATGCGAGGAGATGTC 3' |
| | 5' TCTTTTTACCAATGACAACCTTA 3' |
| H2al | 5' GTTCCTCCATTTATCGTTTCTTCG 3' |
| | 5' GGAGACATTATTTGCCTTTGG 3' |
| H2ag | 5' CTTTGTGGTTGCTCGTAGTGA 3' |
| | 5' TGCCCTTCGCCTTGTGGTG 3' |
| H2am | 5' CACTTTCTGACTTAGGCCACAGGTC 3' |
| | 5' GCCCTTACTTGCCCTTAGCTTT 3’ |
| H2ae | 5' ACAGCAGAAGAACTAACAATCCA 3' |
| | 5' TTGGTTTCTGGGACTCGGGA 3' |
| PRKCE | 5' AGTCAGCACCCACCTCCCC 3' |
| | 5' ATCCTCTTCTCTGTCATTGTGC 3' |
| TGIF2 | 5' GCCCAAGGAGTCGGTGAAGAT 3' |
| | 5' ATGGAGCACACAGACAGGGAGA 3' |

**Supplementary Table S2.** Primers for chromatin immunoprecipitation assay

| **Gene** | | **Sequences** |
|---|---|---|
| *PRKCE* | promoter | 5' GTTCTCGTCCCCGCCCCGTC 3' |
| | | 5' CTAACGGAAAACAGGGAGGGG 3' |
| | 3’UTR | 5' GCTGTTCTTCTGTGTTCTCTCATTAT 3' |
| | | 5' GAAATAACACTAATGAATGGCAAGAG 3' |
| *TGIF2* | promoter | 5' GTCGTAACAAGCGTCTCCATCAG 3' |
| | | 5' CGCTCGGGGGCAGGAAGTG 3' |
| | 3’UTR | 5' GACGACAGCCTTTAGTTTTCCT 3' |
| | | 5' CAGAGGGAGGCAACACAGAG 3' |

| | | |
|---|---|---|
| *c-MYC* | promoter | 5' GCCCTTTCCCCAGCCTTAGC 3' |
| | | 5' ACCATTTTCTTTTGCTCCCT 3' |
| | 3'UTR | 5' CGAGGAGAATGTCAAGAGGCG 3' |
| | | 5' AATCGTTTTCCTTACTTTTCCT 3' |
| | enhancer | 5' GCCAGACAAAATGACAGCCA 3' |
| | | 5' TCAGGTAGGAAGGTTTATTGGATG 3' |
| | 1 | 5' GCCAGACAAAATGACAGCCA 3' |
| | | 5' TCAGGTAGGAAGGTTTATTGGATG 3' |
| | 2 | 5' GCCCTTTCCCCAGCCTTAGC 3' |
| | | 5' ACCATTTTCTTTTGCTCCCT 3' |
| | 3 | 5' AGACGCTGGATTTTTTTCGG 3' |
| | | 5' ACCCCCCACCCCAGCCCCAG 3' |
| | 4 | 5' GCTGCTGCCCACCCCGCCC 3' |
| | | 5'GATGATGATGTTTTTGATGAAGGTC 3' |
| | 5 | 5' TAATGAACTATCTACAAAAATGAGGGGC3' |
| | | 5' CAAGAGGGCGGGGGAAGGAAT 3' |
| | 6 | 5' CGAGGAGAATGTCAAGAGGCG 3' |
| | | 5' AATCGTTTTCCTTACTTTTCCT 3' |
| | 7 | 5' GGGGTGGGAAAGGAGAGAGC 3' |
| | | 5'TTTAGTAGAGACAGGATTTCGCCA3' |
| *BCL2* | 1 | 5' GCTCAGAGGAGGGCTCTTT 3' |
| | | 5' TGCCTGTCCTCTTACTTCATTCT 3' |
| | 2 | 5' GTCTGGGAATCGATCTGGAA 3' |
| | | 5' GCAACGATCCCATCAATCTT 3' |
| | 3 | 5' CACCTGTGGTCCACCTGAC 3' |
| | | 5' CTGAAGAGCTCCTCCACCAC 3' |

| | | |
|---|---|---|
| | 4 | 5' CGCTGCCCGCTTTCTTTCTG 3' |
| | | 5'GCTGTGACTTCCCTGGCCGT 3' |
| | 5 | 5' GGTTAGGGTGACTTTTTGAGGC 3' |
| | | 5' GGGGGATAGAGGAAGGGAGA 3' |
| | 6 | 5' TCGCCCACATCCATAAACCGT 3' |
| | | 5' TCGTCACCGTTTATGCTGCT 3' |
| | 7 | 5' ATCAGAGAAAAACAGGAAAGGCT 3' |
| | | 5' TCCCTCCCACCCCTCCAACA 3' |
| | 8 | 5' AACTCCATCCTGTATCTCTCCTGA3' |
| | | 5' ATGAGCCAACACACCCAGCC 3' |
| | promoter | 5' GCTCAGAGGAGGGCTCTTT 3' |
| | | 5' TGCCTGTCCTCTTACTTCATTCT 3' |
| | 3'UTR | 5' ATCAGAGAAAAACAGGAAAGGCT 3' |
| | | 5' TCCCTCCCACCCCTCCAACA 3' |
| | enhancer | 5' CACCTGTGGTCCACCTGAC 3' |
| | | 5' CTGAAGAGCTCCTCCACCAC 3' |

**Supplementary Table S3.** Primers for 3C assay

| Gene | Sequences |
|---|---|
| *c-MYC* | 5' GAAGGCCCAGGTAAGAAGGAAT 3' |
| | 5' CAACCTCCCTCTCGCCCTAGCC 3' |
| | 5' AGGAAAAGTAAGGAAAACGATT 3' |
| *BCL2* | 5' CCCCCCTGGACCCCCTCTTC 3' |
| | 5' TCTTATTCTGTGGTGTCTTTTGC 3' |
| | 5' CATTTACCACCACATCCTAC 3' |
| | 5' CACCTGTGGTCCACCTGAC 3' |

| | |
|---|---|
| *c-MYC (Input)* | 5' GAGGAGAATGTCAAGAGGCGA 3' |
| | 5' AATCGTTTTCCTTACTTTTCCT 3' |
| *BCL2 (Input)* | 5' CATTTTTTTCTCCTCTTCTTTTTTTTCATTA 3' |
| | 5' ATGTTCTTCTCCTTTTGGGGCTTTTTTTA 3' |
